# Influenza vaccine efficacy induced by orally administered recombinant baculoviruses

**DOI:** 10.1371/journal.pone.0233520

**Published:** 2020-05-27

**Authors:** Swarnendu Basak, Hae-Ji Kang, Su-Hwa Lee, Ki-Back Chu, Eun-Kyung Moon, Fu-Shi Quan

**Affiliations:** 1 Department of Biomedical Science, Graduate School, Kyung Hee University, Seoul, Republic of Korea; 2 Department of Medical Zoology, Kyung Hee University School of Medicine, Seoul, Republic of Korea; 3 Medical Research Center for Bioreaction to Reactive Oxygen Species and Biomedical Science Institute, School of Medicine, Graduate School, Kyung Hee University, Seoul, Republic of Korea; University of South Dakota, UNITED STATES

## Abstract

Although vaccine delivery through the oral route remains the most convenient and safest way for mass immunization purposes, this method is limited by the requirement for large antigen doses and low vaccine efficacy. In this study, we generated recombinant baculoviruses (rBVs) expressing influenza hemagglutinin (A/PR/8/34) and orally delivered a low dose of rBVs to evaluate its vaccine efficacy in mice. Intranasal rBV vaccination was included in the whole experiment for comparison. We found that oral vaccination elicited high levels of virus-specific IgG and IgA antibody responses in both serum and mucosal samples (lung, tracheal, intestinal, fecal and vaginal). Surprisingly, complete protection from the lethal influenza challenge was observed, as indicated by reductions in the virus titer, inflammatory cytokine production, body weight change, and enhanced survival. These results suggest that oral delivery of the influenza rBV vaccine induces mucosal and systemic immunity, which protect mice from the lethal influenza virus challenge. Oral delivery of baculovirus vaccines can be developed as an effective vaccination route.

## Introduction

Influenza is one of the most prevalent vaccine-preventable diseases. Every year, it causes an estimated 3 million cases of illness and from 250,000 to 500,000 deaths throughout the world. Oral delivery of vaccines is the most convenient and safe way of vaccination which could raise immunization coverage [1]. Evidently, the Centers for Disease Control and Prevention has recommended several oral vaccines that are safe, immunogenic, and tolerogenic against cholera [2, 3]. Parenteral vaccination has several issues such as pain associated with needle injection, the requirement for highly trained workers, and opportunistic and iatrogenic infections arising from the use of unsterile needles, as well as the needle-stick injuries, which are especially of high risk in developing countries [4–8]. Importantly, oral vaccination can induce mucosal immunity which might induce protection against influenza infection at the port of entry [1]. Mucosal route-delivered vaccine has been shown to induce higher protection compared to the intramuscular route of administration [9–11]. Importantly, mucosal immunization not only induces mucosal immunity but also able to induce proportionate levels of immune responses at the systemic sites [12–14].

By administering vaccine through the oral route, vaccine particles could easily cross the mucosal barrier through receptor-mediated endocytosis by microfold cells (M cells) of Peyer’s patches, which subsequently results in vaccine particle transcytosis and delivery to the antigen-presenting cells for adaptive immunity generation [7, 13, 15–17]. In fact, most of the mucosal lymphoid tissues are interconnected with each other through the common mucosal immune system throughout the whole body which facilitates antigen-specific immune response induction in the proximal as well as the distal part of the mucosal sites [8]. This could induce virus-specific IgG and IgA antibody responses at all of the mucosal sites such as the lung, mouth, urinary tract, and intestine [1].

Mucosal immune responses can occur at mucosal membranes of the intestines, the urogenital tract, and the respiratory system. As a mucosal immunity for the respiratory system, intranasal administration with the influenza virus has been extensively studied. To date, only a limited number of studies investigating vaccine efficacy induced by orally administered recombinant baculovirus (rBV) vaccine have been conducted. A study has reported that oral immunization with H5N1 hemagglutinin (HA)-expressing live baculovirus could induce high titer of HA-specific IgG and IgA antibodies at systemic as well as mucosal sites [18]. Gastrointestinal route played a critical role in capturing antigens for the large numbers of immune cells residing in the Peyer’s patches [17].

In the present study, rBVs expressing hemagglutinin (A/PR/8/34, H1N1) were generated to evaluate vaccine efficacy in mice, which were orally immunized twice with adjuvant-free rBVs. Intranasal route (IN) immunization was included for vaccine efficacy comparison. We found that oral vaccination induced both mucosal and systemic immunity which were comparable to those induced upon IN vaccination. Oral vaccination elicited virus-specific IgG, IgA antibody responses, significantly reduced lung virus loads, and lessened inflammatory cytokines to result in 100% protection.

## Materials and methods

### Ethics statement

All of the animal experiments have been approved and performed following the Kyung Hee University IACUC guidelines (KHUASP-SE-18-024). Animals were handled by highly trained researchers and maintained under specific pathogen-free conditions with easy access to food and water. Mice were subjected to mild anesthesia using ketamine hydrochloride and xylazine before immunization and bleeding to reduce pain and suffering.

### Cells, viruses, and antibodies

*Spodoptera frugiperda* Sf9 cells cultured using serum-free SF900II medium (Invitrogen, Carlsbad, California, USA) in spinner flasks at 130–135 rpm, 27°C were used for baculovirus production. MDCK cells were cultured in DMEM media supplemented with fetal bovine serum, penicillin, and streptomycin at 37°C with 5% CO_2._ A/Puerto Rico/8/34 (H1N1) viruses were propagated from 11 days old embryonated chicken eggs as described previously [19]. Briefly, allantoic fluid collected from the infected eggs were subjected to sucrose gradient ultracentrifugation for influenza virus purification. Purified virus samples were aliquoted and stored at -80°C until use. Horseradish peroxidase (HRP)-conjugated goat anti-mouse secondary antibodies (IgG, IgG1, IgG2a, IgA) were purchased from Southern Biotech (Birmingham, AL, USA).

### Cloning, recombinant baculovirus generation, and baculovirus plaque assay

Recombinant baculoviruses were produced as previously described [19]. Briefly, A/PR/8/34 (H1N1) HA gene was cloned into pFastBac plasmid and transformed into DH10Bac competent cell. Transformant DNA was transfected into Sf9 cells for rBV production. Sf9 cells were seeded into a 96-well plate for baculovirus plaque assay. Cells were incubated with serially diluted viruses for 1 hour and upon virus removal, cells were overlayed with 0.8% noble agar and incubated for 3 days at 37 °C. After overlay removal and fixation, cells were blocked with 3% skim milk then incubated with an anti-HA antibody. HRP-conjugated mouse IgG was used as secondary antibody and 3, 3-diaminobenzidine (DAB) chromogenic substrate was used to visualize plaques, which were counted under the microscope.

### Hemagglutination (HA) assay

A/PR/8/34 (H1N1) HA-expressing baculoviruses were serially diluted and added into a round-bottom 96-well plate. After the addition of 0.5% chicken red blood cells (RBCs), plates were incubated at room temperature for 1 hour and HA titer was determined. Vaccine immunization dosage was determined based on the plaque assay and HA titer results.

### Immunization and challenge

Twenty-four seven-week-old female BALB/c mice were purchased from NARA Biotech (Seoul, South Korea). Groups of mice (n = 6 per group) were prime and boost immunized at weeks 0 and 5 through oral or intranasal route. Animals were starved for 2 h and were immunized intragastrically by oral gavage twice at 4 week intervals in 100ul PBS containing rBV-HA (6x10^6^ pfu). Four weeks after boost immunization, mice were intranasally challenged with 125 pfu/ml (5LD_50_) of A/PR/8/34 (H1N1) virus in 100 μl. Mice were monitored on a daily basis for up to 14 days to measure body weight changes and survival rates. Mice whose bodyweight loss exceeded 25% of its initial value were humanely euthanized.

### Sample collection and preparation

Blood samples from all of the groups were collected 4 weeks after prime and boost immunization through retro-orbital plexus puncture. Sera were separated from blood by centrifugation at 2000 rpm for 10 min at 4°C and stored at –20°C until use. At day 4 post-challenge, half of the mice from each group were randomly selected and sacrificed for sample collection. Trachea, lung, intestine, spleen, bone marrow, vaginal secretions, and feces were collected and processed individually for further analysis of several immunological parameters as described before [1, 19–22].

### Virus-specific antibody responses

Influenza A virus A/PR/8/34 (H1N1)-specific IgG, IgG1, IgG2a, and IgA responses were measured by enzyme-linked immunosorbent assay (ELISA) following the protocol previously described [19]. After coating 96-well plates overnight at 4 °C with 4 μg/ml of inactivated A/PR/8/34 antigen, plates were blocked with 0.05% FBS in PBST for 1.5 hr at 37°C. After incubating with diluted sera for 1 hr at 37 °C, wells were washed and incubated with HRP-conjugated goat anti-mouse secondary antibodies. O-phenylenediamine dissolved in citrate-phosphate buffer with H_2_O_2_ was used as substrate and reactions were stopped with 2N H_2_SO_4_. Antibody titer, defined as the reciprocal of the highest dilution that still gives positive results (with the cutoff OD value being twice that of the background OD value), were determined by ELISA as previously described [23, 24]. Colorimetric changes were assessed by measuring the optical density values at 450 nm using EZ Read 400 microplate reader (Biochrom Ltd., Cambridge, UK).

### Lung viral titers, cytokine, and antibody-secreting cell (ASC) responses

Lung viral titer was determined using the method previously described [20]. Briefly, lung tissues were individually homogenized in 1ml PBS and passed through a cell strainer to remove tissue debris. Homogenates were centrifuged for 20 minutes at 3000 rpm, 4°C, and the supernatants were stored at -80°C until use. Serially diluted lung supernatants were inoculated into a confluent monolayer of MDCK cells and incubated for 2–3 days at 37°C with 5% CO_2_. Following incubation, cells were fixed with 0.25% glutaraldehyde and stained with 1% crystal violet for plaque visualization. Interferon-gamma (IFN-γ) and interleukin-6 (IL-6) responses from the lung supernatants were measured using the BD OptEIA ELISA Kit (BD Biosciences, San Jose, CA, USA) as previously described [20]. Antibody-secreting cell assay was performed using isolated splenocytes and bone marrow cells as previously described [19, 25]. Briefly, 96-well culture plates (SPL Life Sciences, Pocheon, South Korea) were coated overnight with inactivated influenza A/PR/8/34 (H1N1) virus (4ug/ml) and after blocking with 0.5% bovine serum albumin, isolated splenocytes and bone marrow cells were added to each well. Plates were incubated for 4 days at 37°C with 5% CO_2_ and subsequently incubated with HRP-conjugated goat-anti mouse IgG antibody for 1 h at 37°C.

### Preparation of splenocytes and lung cell for flow cytometry

Single cell suspension of splenocytes was acquired to measure the B cell population using the method previously described [20]. Individual spleens were homogenized and RBCs were removed using RBC lysis buffer (Sigma-Aldrich, St. Louis, MO, USA) for the acquisition of single cell splenocyte suspension. After cell counting with a hemocytometer, cell suspensions were stimulated with inactivated A/PR/8/34 (H1N1) virus at a concentration of 4 μg/ml for 4 hr at 37 °C with 5% CO_2_. Cells were centrifuged and resuspended in staining buffer (2% bovine serum albumin and 0.1% sodium azide in 0.1 M PBS) along with Fc block (clone 2.4G2; BD Biosciences, CA, USA) and incubated at 4 °C for 15 min. After staining the cells with B220-FITC, and GL7-PE (BD, San Diego, CA, USA) fluorophore-conjugated antibodies, cells were incubated at 4°C for 30 min. Cells were acquired and analyzed using BD C6 Accuri flow cytometer (BD Biosciences, San Jose, CA, USA).

### Statistical analysis

All parameters were recorded for individuals within all groups. One-way ANOVA with Tukey’s *post hoc* test or 2-way ANOVA with Bonferroni’s *post hoc* test were performed on all sets of data to determine statistical significance using GraphPad Prism version 5 (San Diego, CA, USA). The data was considered to be statistically significant if *P* value * < 0.05, ** < 0.01, *** < 0.001.

## Results

### Generation of recombinant baculovirus and HA titer

**Fig 1:**
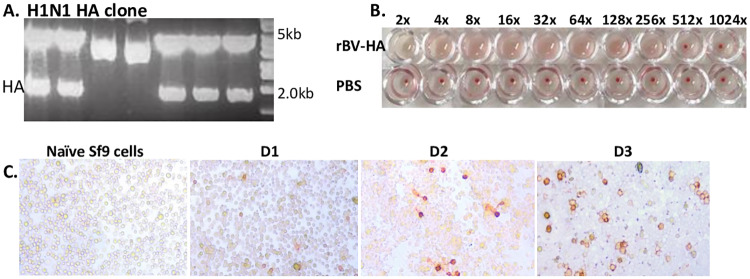
Gene cloning, HA titer for baculovirus, and plaque assay. After restriction enzyme digestion, A/PR/8/34 (H1N1) incorporation of HA gene into the pFastBac vectors was confirmed. (A) Upon enzymatic cleavage, transformants containing the HA gene within pFastBac vectors were selected. (B) HA titers of baculoviruses expressing the HA were determined. The titers were calculated to be 1:256. (C) Sf9 cells were infected with baculovirus expressing H1N1-HA. No plaques were detected in the naïve control, whereas plaque formation was clearly visible from day 1 onwards.

Constructs containing influenza HA from A/Puerto Rico/8/34 are shown in Fig 1. Incorporation of HA gene into the pFastBac vector was confirmed through restriction enzyme digestion followed by agarose gel electrophoresis (Fig 1A). HA titer of the recombinant baculoviruses expressing influenza HA was deternubed to be 256 titer (Fig 1B). Recombinant baculoviruses titers were quantified using a plaque assay (Fig 1C). There were no significant differences in plaque formation between day 3 and day 4.

### Oral vaccination induces humoral immune response comparable to those induced by IN vaccination

**Fig 2:**
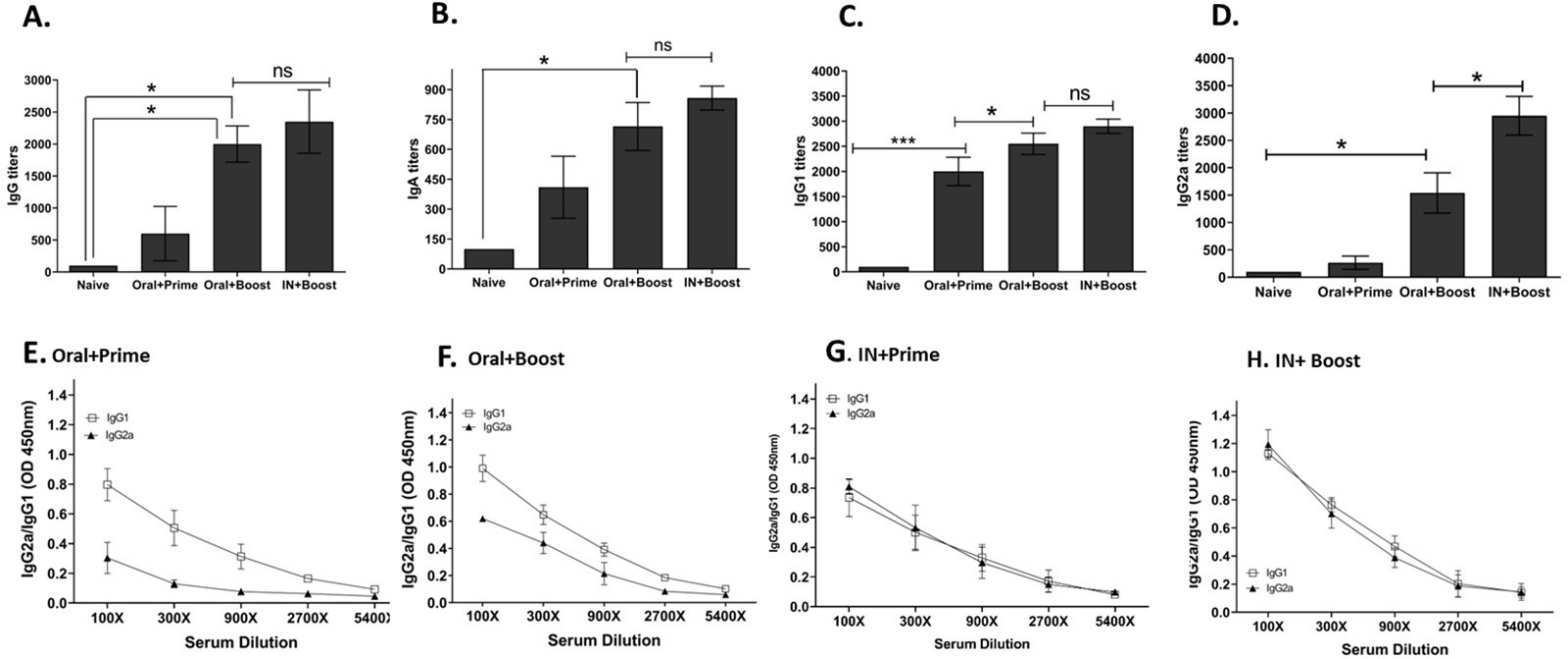
Influenza A/PR8 virus-specific serum antibody responses. Groups of mice (n = 6 per group) were prime and boost immunized through either oral or intranasal route with rBV-HA. Sera were collected at 4 weeks after prime and boost (B), After challenge infection, sera were collected and IgG (A), IgA (B), IgG1 (C), and IgG2a (D) titers were measured. All of the antibody titers were drastically enhanced upon boost immunization. IgG1 and IgG2a antibody titers from oral and intranasal route immunization after prime and boost were compared (E-H). Data are expressed as mean ± SEM. Statistical significances were denoted using *P < 0.05 and ***P < 0.001, whereas “ns” was used to indicate no statistical significance.

Orally immunized mice exhibited substantially higher level of A/PR8-specific serum IgG, IgA, IgG1 and IgG2a antibody titers after prime and boost compared to the naïve (Fig 2A–2D). In comparison to the IN-immunized mice, no significant differences in IgG, IgA, and IgG1 titers were observed after boost immunization (Fig 2A–2C). However, significant differences in serum IgG2a titers were observed between orally immunized and intranasally immunized mice groups (Fig 2D). Comparison of IgG1 and IgG2a titers following prime and boost immunization through the oral route indicated that vastly greater quantities of IgG1 were produced compared to IN route immunization (Fig 2E–2H).

### Oral vaccination induces antibody-secreting cell responses

**Fig 3:**
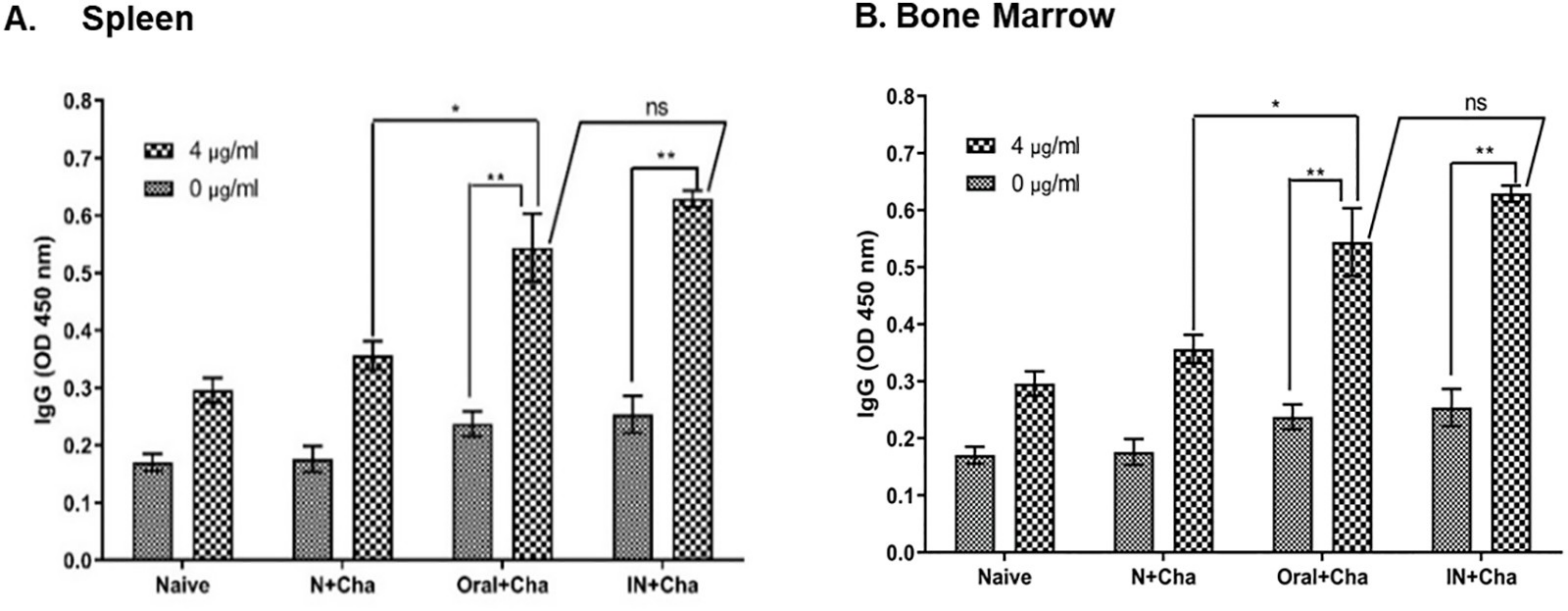
Antibody-secreting cells (ASC) response induced by orally immunized rBV. Single cell suspension of the spleen (A) and bone marrow (B) cells were either unstimulated or stimulated with 4 μg/ml of inactivated PR8 virus. ASC responses in unstimulated cells were relatively similar for all groups, whereas significantly higher ASC responses were observed in Oral+Cha mice groups in both spleen and bone marrow compared to non-immunized control upon antigen stimulation. There were no significant differences between ASC responses of stimulated Oral+Cha and IN+Cha groups. Data are expressed as mean ± SEM. Statistical significances were denoted using *P < 0.05 and **P < 0.01, whereas “ns” was used to indicate no statistical significance.

To evaluate the vaccine efficacy of orally inoculated rBVs, spleen and bone marrow cells of mice were collected and cultured for 4 days. As expected, oral immunization conferred higher IgG antibody responses from both splenocytes and bone marrow cells compared to naïve or naïve-challenge groups (Fig 3A and 3B). Additionally, no significant differences in IgG levels were observed between oral and IN immunized mice.

### Mucosal immunity induction upon oral vaccination

**Fig 4:**
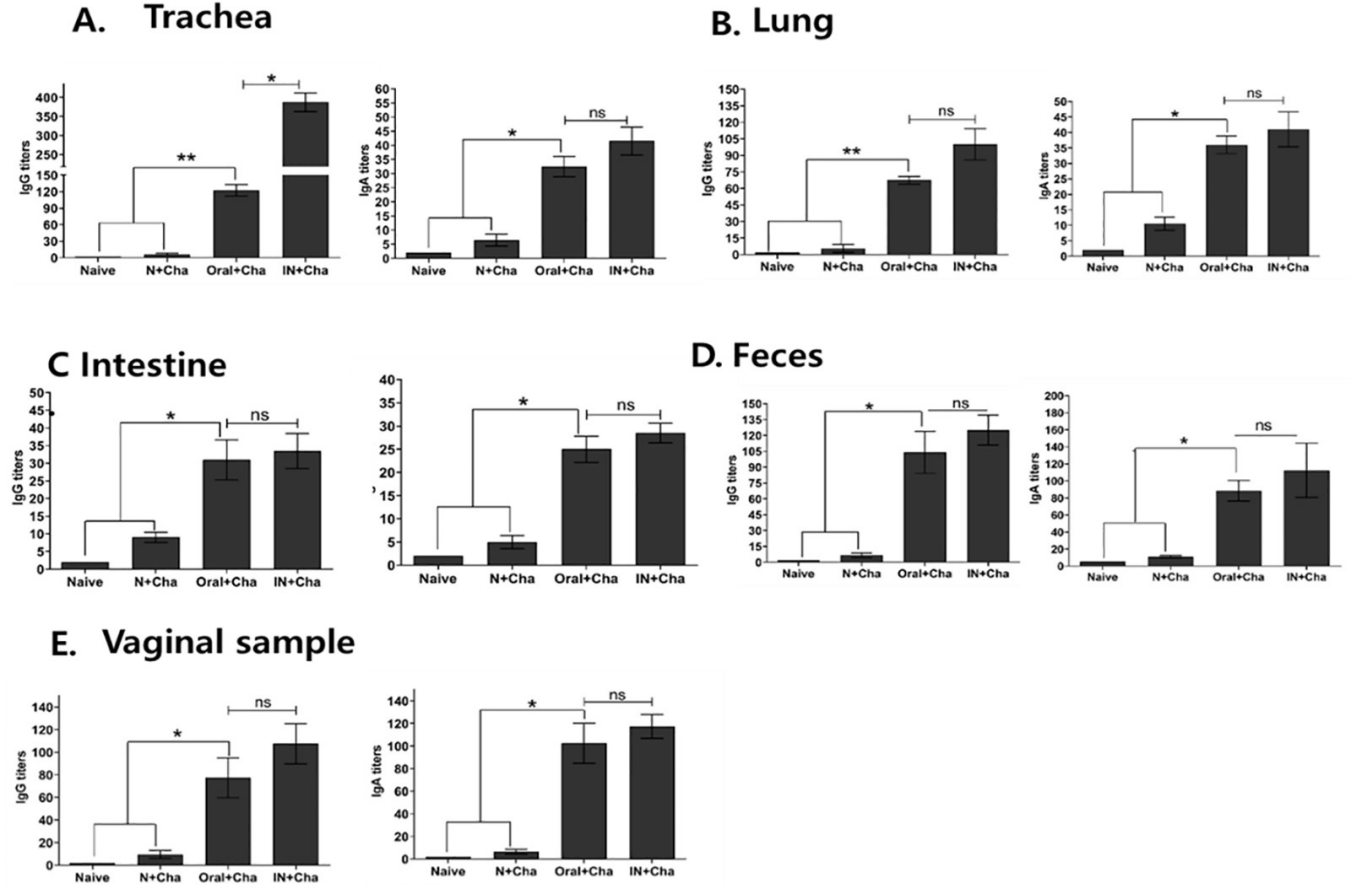
Influenza A/PR8 virus-specific mucosal IgG as well as IgA responses. Antibody titers from mucosal samples including trachea (A), lung (B), intestine (C), feces (D), and vaginal secretion (E) were determined using ELISA. Data are expressed as mean ± SEM. Statistical significances were denoted using *P < 0.05 and **P < 0.01, whereas “ns” was used to indicate no statistical significance.

To determine whether enhanced mucosal immunity was induced by oral vaccination, virus-specific IgG and IgA titers from the trachea, lung, intestine, feces, and vaginal secretions were assessed post-challenge infection (Fig 4). High titer of IgG antibody response was observed in all of the mucosal samples (trachea, lung, intestine, vagina, feces) from oral immunized mice group after challenge. Although significant differences in IgG titers between oral and IN immunized mice were noticeable in trachea and lung samples (Fig 4A and 4B), such differences were not present in intestinal, fecal, and vaginal samples (Fig 4C–4E). IgA titers from all five mucosal sites were induced at substantial levels, albeit much less compared to IgG. In contrast to IgG tracheal and lung samples, significant differences between the two immunization methods were not observed.

### Oral immunization induces germinal center B cell response

**Fig 5:**
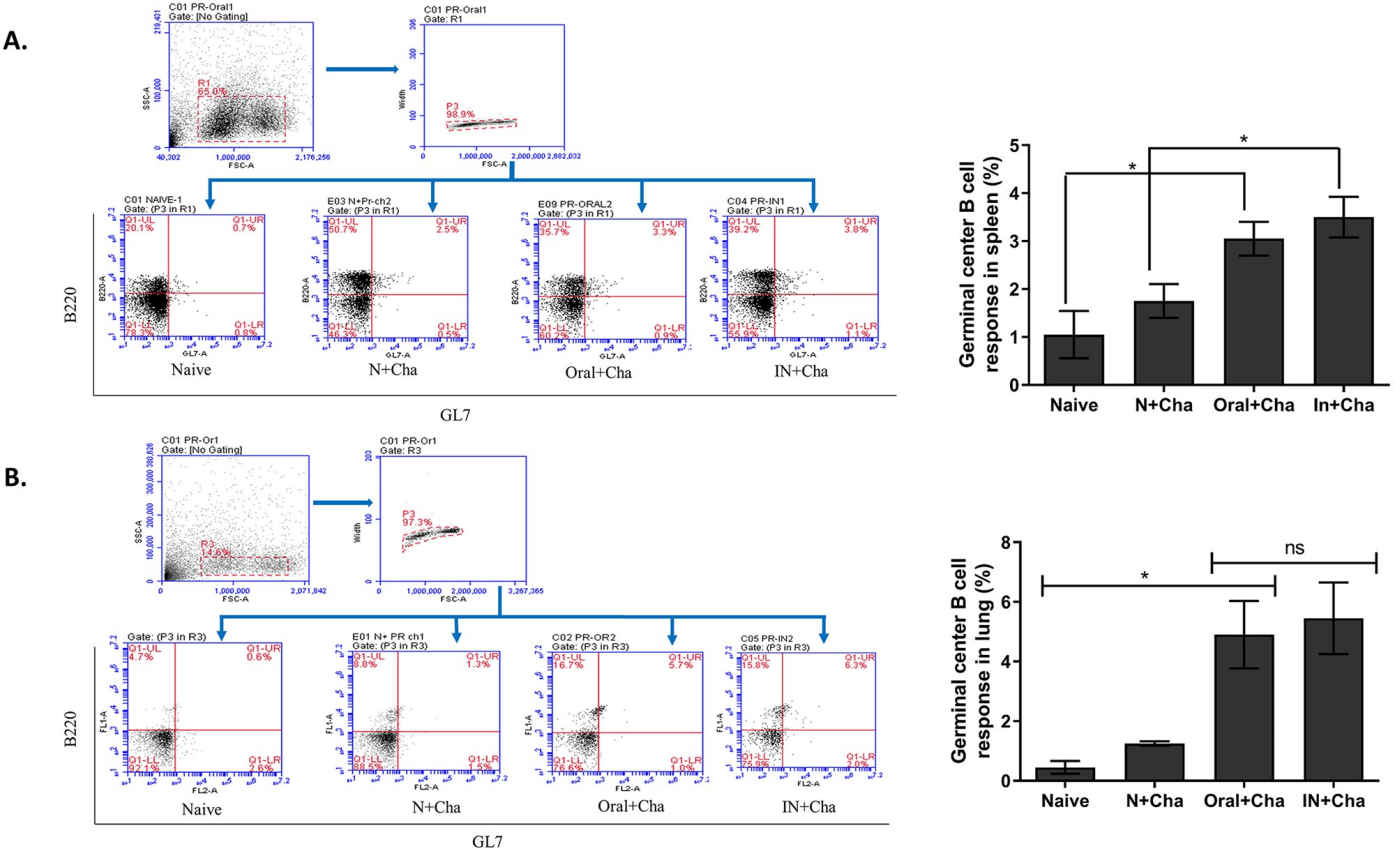
Oral vaccination induces germinal center B cell response in the spleen and lung. Flow cytometry plots showing the gating strategy to identify germinal center cells in spleen (A) and lung (B) cells. Higher populations of GC B cells were detected in the lung sample of Oral+Cha group compared to Naive or N + Cha group. Data are expressed as mean ± SEM. Statistical significances were denoted using *P < 0.05, whereas “ns” was used to indicate no statistical significance.

Lymphocyte populations from spleen and lung were gated and germinal center (GC) populations were measured using fluorescence-acquired cell sorting. Oral immunization elicited a higher percentage of GC B cell response in both spleen and lung compared to naïve and naïve-challenge groups (Fig 5A and 5B). No significant difference was observed between oral and IN immunized mice groups.

### Oral immunization significantly reduced lung inflammatory responses

**Fig 6:**
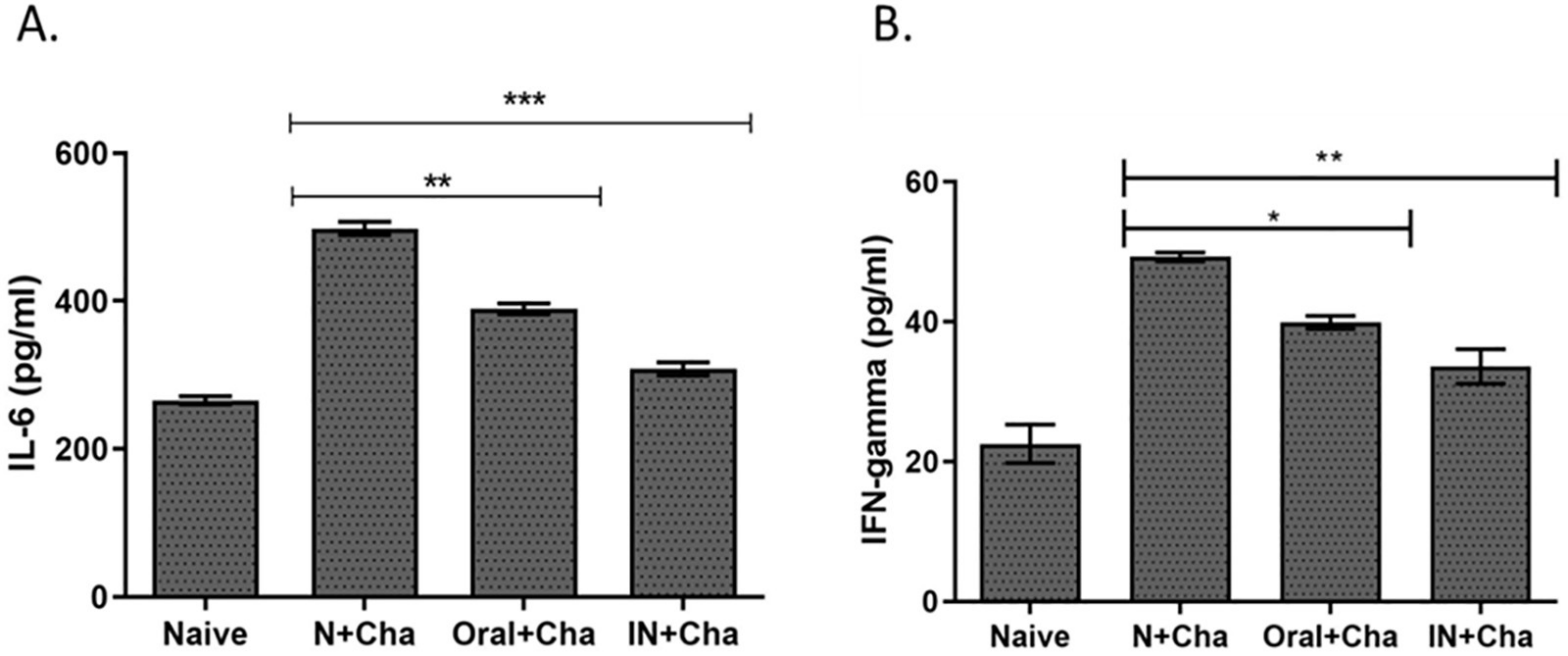
Pro-inflammatory cytokine (IFN-γ and IL-6) responses in the lung. Lungs from individual mice in each group were collected on day 4 post-challenge to assess IFN-γ (A) and IL-6 (B) responses. Data are expressed as mean ± SEM. Statistical significances were denoted using *P < 0.05, **P < 0.01, ***P < 0.001, whereas “ns” was used to indicate no statistical significance.

At 4 days post-infection (dpi), lung samples were isolated to measure the concentration of inflammatory cytokines IFN-γ and IL-6. As expected, lesser quantities of pro-inflammatory cytokines were detected from immunized mice (Fig 6A and 6B). Furthermore, differences in both IFN-γ and IL-6 cytokine responses between the two immunized groups were negligible.

### Oral immunization confers protection against lethal challenge infection

**Fig 7:**
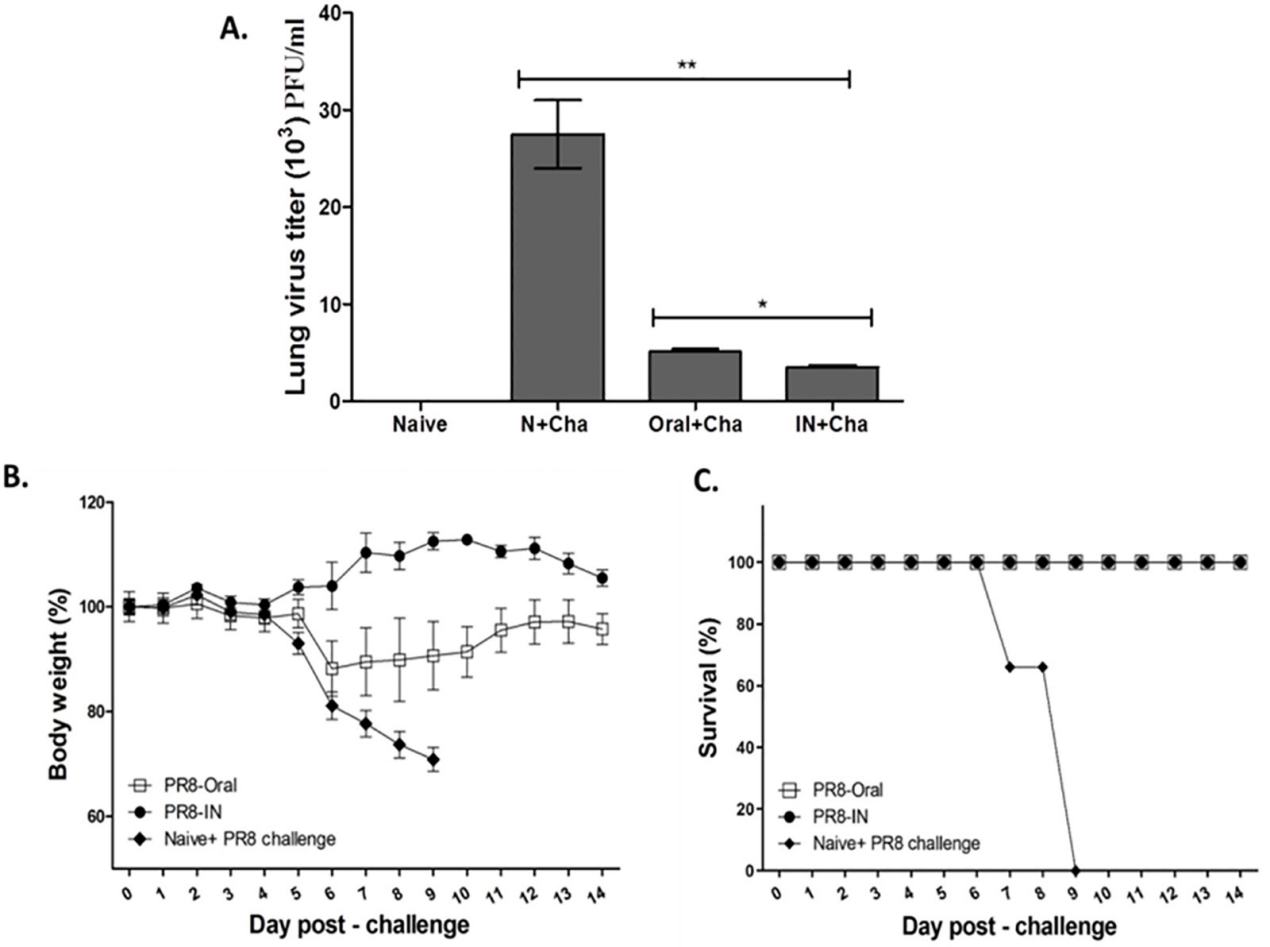
Lung virus titers and body weight changes and survival. Mice were challenged with a lethal dose (A/PR8 virus, 5LD_50_) after boost immunization. (A) Lung homogenates were collected 4 dpi and viral titers were calculated using plaque assay. Lower virus titers were detected in both Oral+Cha and IN+Cha groups compared to the N + Cha group. Mice were monitored daily for body weight (B) and survival (C) measurement. Data are expressed as mean ± SEM. Statistical significances were denoted using *P < 0.05 and **P < 0.01.

Mice were sacrificed 4 dpi and the lung homogenates were used to determine lung viral titer. Significant differences in lung viral titers between immunized mice and control mice were observed post-challenge infection (Fig 7A). As expected, oral immunization lessened the lung viral titer compared to the infection control group. Similarly, IN immunization also demonstrated significantly less lung viral titer compared to both infection control and oral immunization. Noticeable bodyweight reduction was observed at 5 dpi from both oral immunization and naïve + challenge mice (Fig 7B). Drastic bodyweight loss for naïve + challenge mice continued onwards to 9 dpi and mice were euthanized as humane intervention endpoint has been reached. Bodyweight recovery for the orally immunized mice occurred from 6 dpi. Vaccinated mice demonstrated 100% survival, confirming complete protection against lethal challenge infection, whereas all of the naïve + challenge mice perished by 9 dpi (Fig 7C).

## Discussion

Mucosal immunity can induce protection at mucosal sites upon pathogen entry. Oral and intranasal vaccines are the two main options for mucosal administration. However, intranasal vaccines have adverse side effects including asthma, reactive airway disease, and other chronic pulmonary or cardiovascular disorders [26]. Therefore, oral vaccines seem to be the safest alternative [27].

Oral delivery of inactivated whole influenza virus vaccines has been recognized as an effective vaccination route [1]. Oral vaccination of mice with inactivated whole influenza vaccine induces systemic and mucosal immunity, providing complete protection against homologous or heterologous challenge infections. However, higher doses or multiple vaccinations are needed since gastric fluids reduce the stability of orally delivered vaccines which decreases vaccine antigenicity [1].

Clinical trials using recombinant baculovirus-expressed hemagglutinin influenza vaccine have confirmed its safety and immunogenicity upon parenteral immunizations [28, 29]. Full length of HA expressed in oligomeric form elicited the strongest immune response in mice, and orally delivered HA displaying baculovirus vaccine protected mice against H5N1 infection [18].

In this study, we have assessed the baculovirus vaccine efficacy against influenza A (H1N1) virus. Intranasally immunized mice with the same dose were included to compare the vaccine efficacy induced by the oral route of immunization. Intranasal immunization of rBV vaccine expressing HA (A/California/04/09, 3×10^7^pfu) conferred complete protection against homologous as well as heterologous virus challenge infections [30]. Gastrointestinal delivery of live recombinant baculovirus expressing H5N1 haemagglutinin (BacHA) was able to significantly induce systemic IgG response and IgA response at proximal mucosal sites such as the intestines, resulting in complete protection after 3 immunizations [18]. High doses of recombinant baculoviruses were also used to induce protection, although the mucosal aspect of immunity has not been analyzed in detail [26]. In our present study, low doses (1x10^6^ pfu) of rBVs were used for oral immunization which was able to induce systemic and mucosal IgG and IgA antibody responses after 2 immunizations, providing complete protection. In our study, orally immunized mice showed higher levels of virus-specific IgG1 antibody response compared to the IgG2a antibody subtype. Consistent with the previous report, similar titers of IgG1 and IgG2a antibodies were observed from intranasally immunized mice, indicating balanced Th1 and Th2 immune responses [31]. Here, we report that oral immunization with rBV-HA can induce significantly higher levels of GC B cell and antibody-secreting cell responses. Our result showed that orally immunized mice showed higher antibody secreting cell response in the spleen and bone marrow, in addition to heightened GC B cell response in both the spleen and the lung. Miniscule amounts of GC B cell and ASC responses were detected from unvaccinated naïve mice post-challenge. Our results indicate that oral vaccination can induce antibody-secreting plasma cell responses at an early time point post-challenge. As such, the memory B cells generated in response to rBV-HA oral vaccination enables differentiation into antibody-secreting plasma cells during pathogen re-encounter. From this, it is evident that oral delivery of rBV-HA vaccine activates antigen-presenting cells such as dendritic cells (DCs). In addition, the delivery of HA protein into the intestinal epithelial cells results in its uptake by M cells through receptor-mediated endocytosis, which is subsequently delivered to the DCs for antigen cross-presentation via MHC-II dependent pathway for GC induction [32, 33]. Eventually, the induction of antigen-specific GC B cells leads to the release of memory B cell and long-lived antibody-secreting plasma cell at the systemic sites to assist in viremia reduction.

Our present study showed that orally immunized mice possessed significantly reduced lung inflammatory cytokines such as IL-6 and IFN-γ after challenge infection with lethal influenza H1N1 (A/PR/8/34) compared to non-immunized control mice. As expected, all of the vaccinated mice showed low lung viral titers. This result is also consistent with the body weight and survival data. All orally immunized mice were 100% protected against 5LD_50_ dose of A/PR/8/34 (H1N1) virus. Identical results were also observed from intranasally immunized mice, whereas all of the unvaccinated mice were dead by 9 dpi. Similarly, bodyweight recovery for all of the immunized mice occurred from day 5 onwards and eventually, complete recovery was observed by day 14.

## Conclusion

Overall, oral route is an effective route for baculovirus vaccine administration that induces humoral immunity at the systemic and mucosal immunity which ensures rapid recovery and protection against lethal H1N1 influenza virus infection. Elevated level of GC B cell and antibody-secreting cell responses were induced by the oral route of vaccination with rBV-HA. Our study provides insightful knowledge for designing and constructing an effective oral recombinant baculovirus vaccine against influenza A virus. Oral delivery is a viable option for vaccination due to its increased patient compliance, safety, and ease of administration. The strategy for the vaccine strain selection, vaccine design, and route of administration described herein will provide an idea for the development of a broadly protective vaccine against influenza virus infection.

## Supporting information

S1 Raw images (PDF)

S1 Checklist (DOCX)
