# Supplementary figures and images for: Influenza vaccine efficacy induced by orally administered recombinant baculoviruses
Source: PLoS One. 2020 May 27;15(5):e0233520. doi: 10.1371/journal.pone.0233520 (PMC7252623; doi:10.1371/journal.pone.0233520)

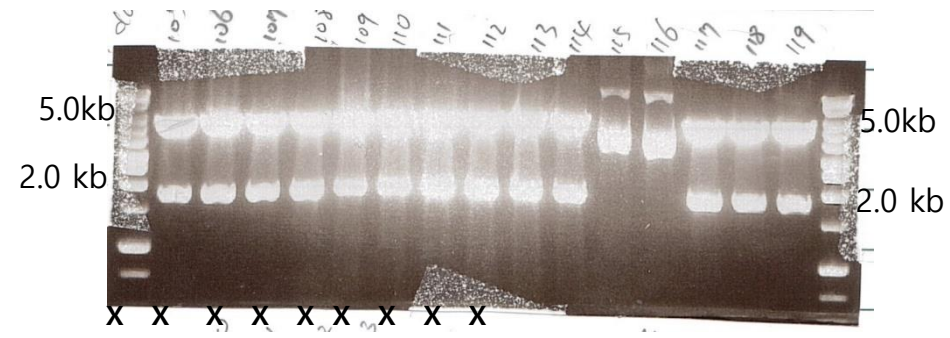

Supplement: S1 Raw images — (PDF) [file pone.0233520.s001.pdf]
